# Comparing clinician descriptions of frailty and geriatric syndromes using electronic health records: a retrospective cohort study

**DOI:** 10.1186/s12877-017-0645-7

**Published:** 2017-10-25

**Authors:** Laura J. Anzaldi, Ashwini Davison, Cynthia M. Boyd, Bruce Leff, Hadi Kharrazi

**Affiliations:** 1 Department of General Internal Medicine, Johns Hopkins University School of Medicine, 624 N Broadway, Baltimore, MD 21205 USA; 2 Center for Population Health IT, Department of Health Policy and Management, Johns Hopkins Bloomberg School of Public Health, Baltimore, MD USA; 3 Center for Transformative Geriatric Research, Division of Geriatric Medicine and Gerontology, Johns Hopkins University School of Medicine, Baltimore, MD USA

**Keywords:** Frailty, Geriatric syndromes, Electronic health records, Unstructured data, Natural language processing

## Abstract

**Background:**

Geriatric syndromes, including frailty, are common in older adults and associated with adverse outcomes. We compared patients described in clinical notes as “frail” to other older adults with respect to geriatric syndrome burden and healthcare utilization.

**Methods:**

We conducted a retrospective cohort study on 18,341 Medicare Advantage enrollees aged 65+ (members of a large nonprofit medical group in Massachusetts), analyzing up to three years of administrative claims and structured and unstructured electronic health record (EHR) data. We determined the presence of ten geriatric syndromes (falls, malnutrition, dementia, severe urinary control issues, absence of fecal control, visual impairment, walking difficulty, pressure ulcers, lack of social support, and weight loss) from claims and EHR data, and the presence of frailty descriptions in clinical notes with a pattern-matching natural language processing (NLP) algorithm.

**Results:**

Of the 18,341 patients, we found that 2202 (12%) were described as “frail” in clinical notes. “Frail” patients were older (82.3 ± 6.8 vs 75.9 ± 5.9, *p* < .001) and had higher rates of healthcare utilization, including number of inpatient hospitalizations and emergency department visits, than the rest of the population (*p* < .001). “Frail” patients had on average 4.85 ± 1.72 of the ten geriatric syndromes studied, while non-frail patients had 2.35 ± 1.71 (*p* = .013). Falls, walking difficulty, malnutrition, weight loss, lack of social support and dementia were more highly correlated with frailty descriptions. The most common geriatric syndrome pattern among “frail” patients was a combination of walking difficulty, lack of social support, falls, and weight loss.

**Conclusions:**

Patients identified as “frail” by providers in clinical notes have higher rates of healthcare utilization and more geriatric syndromes than other patients. Certain geriatric syndromes were more highly correlated with descriptions of frailty than others.

**Electronic supplementary material:**

The online version of this article (10.1186/s12877-017-0645-7) contains supplementary material, which is available to authorized users.

## Background

Geriatric syndromes are multifactorial conditions, common in older adults [1], and associated with poor outcomes such as morbidity, hospitalizations, and nursing home admissions [2]. A high burden of geriatric syndromes may contribute to frailty, which is an overarching phenotype of vulnerability in older adults [1, 3, 4]. Frailty is related to, but distinct from, comorbidity and disability [3]. There are two prevailing validated models of frailty: Fried et al.’s syndromic model and Rockwood et al.’s accumulation of deficits model [5]. The syndromic model is categorical, such that the presence of 3 or more of the following defines frailty: impaired mobility, slow walking speed, unintentional weight loss, weak grip strength, and low physical activity [6]. The accumulation of deficits model creates a continuous frailty index and is based on a predefined set of signs, symptoms, comorbidities and disabilities [7]. The estimated prevalence of frailty in older adults ranges widely from 4% to 59% depending on the definition and the population studied [3].

Measuring frailty at a population level is of interest to healthcare providers as frailty is highly associated with morbidity, mortality, and adverse health outcomes. This has led to the increased recognition of frailty in primary care and incentivizing the encoding of frailty in electronic health records (EHR) internationally [8, 9]. For example, Clegg et al. have developed an electronic frailty index built from the accumulation of deficits model using Read-CTV3 codes in the U.K. [10]. However, most EHR systems in the U.S. use the international classification of disease (ICD) standard for diagnostic codes [11], which does not have an accepted code for “frailty” yet [12, 13]. Given the limitations of ICD-based EHRs and lack of incentives to encode frailty in the U.S., this research has studied the availability and specification of frailty wording in EHR’s free text, where clinicians can describe a patient as “frail” [14]. Using a previously developed and validated natural language processing (NLP) algorithm on clinical notes from a large population, we were able to identify patients whom healthcare providers labelled in the EHR as “frail”. Our study aimed to determine which geriatric syndromes were most associated with provider descriptions of frailty, and how patients described as “frail” differed from other older adults in terms of geriatric syndrome burden and healthcare utilization.

## Methods

This is a retrospective cohort study that used claims and EHR data from Atrius Health, a nonprofit medical group based in eastern Massachusetts and a Pioneer accountable care organization (ACO) and CMS Next Generation participant [15]. We studied a cohort of Atrius Health members aged 65 or older who received health insurance coverage through a local Medicare Advantage plan. As Atrius Health accepts full financial risk for this population, administrative claims data included care received at any setting or location that was reimbursable under the Medicare Advantage plan.

EHR data included both structured fields and unstructured clinical notes (“free text”), and was limited to providers using the medical group’s specific EHR system. Clinical notes included documentation from the following settings: outpatient visits, home-care/nursing visits, emergency room visits, discharge summaries, and patient e-mails and phone calls. Inpatient hospitalization records were represented incompletely in the outpatient EHR data, thus were removed to reduce potential data quality biases [16, 17].

Structured EHR data was obtained in a HIPAA compliant format [18]. Protected health information was scrubbed from both structured and unstructured data prior to the analysis. All data was stored on a secured network approved by the institutional review board of Johns Hopkins University (IRB# 6196).

### Population

We received data collected between January 1, 2011 and December 31, 2013 on 20,347 Atrius Health members aged 65 or older. Of the total patients, 18,635 met criteria to have 13 months of continuous enrollment in the ACO during the study period. Our analysis focused on the 18,341 patients who had at least one free text note during their first 12 months of continuous enrollment. Sensitivity analysis of the excluded population did not reveal any significant differences.

### Constructs of geriatric syndromes

An expert panel made up of four physicians, including two academic geriatricians, used consensus judgement to select and operationalize ten geriatric syndromes: falls, malnutrition, dementia, severe urinary control issues, absence of fecal control, visual impairment, walking difficulty, pressure ulcers, lack of social support, and weight loss. The same expert panel generated a list of diagnosis codes (international classification of diagnosis; ICD) corresponding to each syndrome to query the claims and structured EHR fields, and a seed list of phrases that, if present in the free text, would indicate the presence of that syndrome.

### Natural language processing algorithm

We used a pattern-based natural language processing (NLP) algorithm to parse EHR free text and identify the presence of each of the ten syndromes as well as explicit mention of frailty. Using the seed phrases described above as a guideline, a team of three reviewed 185 patient records and manually identified additional phrases in the free text that corresponded to each syndrome. Explicit observations that a patient was frail were also identified. A sample of identified phrases from each syndrome as well as mentions of frailty is available in online Additional file 1.

The final list of phrases was used to develop a list of regular expressions (a specific syntax used by many programming languages to describe search patterns) which were used to automatically identify the syndromes and mentions of frailty in the EHR’s free text. Regular expression patterns were refined via multiple iterations of manual review. Once the algorithm was finalized, the records of 100 randomly selected patients flagged for each syndrome were reviewed to approximate false positive rates in the full population of 18,341. NLP syndrome identification was considered to be a false positive if, upon manual review, the algorithm found a phrase that did not indicate the presence of the assigned construct. False positive rates for the syndromes ranged from 1% to 15%. For frailty, manual review of 164 random samples revealed a false positive rate of 3%. Additional details on the methodology and evaluation of our regular expression NLP algorithm are described in online Additional file 1.

### Case identification

We ran the NLP algorithm described above on all available notes of all 18,341 patients. A patient was labeled with a geriatric syndrome if (1) the algorithm identified at least one positive phrase corresponding to that syndrome in any of the patient’s unstructured EHR notes, or if (2) at least one of the expert-panel-generated ICD codes was found in the patient’s structured data. We flagged a patient as “frail” if the algorithm found one or more explicit mentions of frailty in any of the patient’s free text notes. We did not require that geriatric syndromes be found in the notes generated within the same clinical visit as evidence of frailty due to potential inaccuracies of extracting temporal data from free text.

### Statistical analysis

Statistical analysis included data stratification, descriptive statistics, linear regression (variable adjustment), and Pearson correlation. Analysis was performed in Microsoft Excel® (2016) and R (version 3.2.3).

## Results

### Population

The population denominator included 18,341 patients with a mean age of 75.9 years and 58.9% females (Table 1). Johns Hopkins ACG® Software (version 10) [19] was used to derive healthcare utilization rates from administrative claims data. The average number and length of free text notes for each patient were 132 notes and 165,766 characters. There were 2202 patients with frailty mentions (12% of the full population). These patients were significantly older (mean age of 82.3) and more likely to be female (66.5%). In comparing the average rates of healthcare utilization, we found that “frail” patients had 3.5× more inpatient hospitalizations, 2.4× more emergency department visits and 5.1× more readmissions compared to patients without a frailty label (*p* < .001). When adjusting the analysis for age and sex, with and without the number of comorbidities, we found that the mean of a frailty label was 1.40× and 2.26× higher for inpatient hospitalizations, 1.34× and 1.86× more for emergency department visits, and 1.41× and 2.71× higher for readmissions compared to the non-frail population (Table 1).

**Table 1:** Population Demographics and Healthcare Utilization*

Demographics/Utilization^a^	Full Population	“Frail” Population	“Non-Frail” Population
Population (N)	18,341	2202	16,139
Age in years (SD)^b^	75.9 (7.5)	82.3 (6.8)	75.1 (7.2)
Sex (%)^b^	F 10,806 (58.9) M 7535 (41.1)	F 1464 (66.5) M 738 (33.5)	F 9342 (57.8) M 6797 (42.2)
Average comorbidity count (SD)^b^	9.16 (4.72)	13.19 (5.23)	8.61 (4.37)
Average medication ingredients (SD)^b^	12.57 (7.73)	16.81 (8.94)	12.00 (7.36)
Average IP events (SD)^b^	0.86 (1.59)^c^ 0.86 (0.35)^d^ 0.86 (0.91)^e^	2.28 (2.67)^c^ 1.15 (0.32)^d^ 1.69 (0.97)^e^	0.66 (1.26)^c^ 0.82 (0.34)^d^ 0.75 (0.84)^e^
Average ED events (SD)^b^	0.62 (1.26)^c^ 0.62 (0.21)^d^ 0.62 (0.44)^e^	1.25 (2.18)^c^ 0.80 (0.19)^d^ 1.05 (0.45)^e^	0.53 (1.04)^c^ 0.60 (0.20)^d^ 0.56 (0.41)^e^
Average readmission events (SD)^b^	0.15 (0.65)^c^ 0.15 (0.06)^d^ 0.15 (0.22)^e^	0.51 (1.30)^c^ 0.20 (0.06)^d^ 0.33 (0.24)^e^	0.10 (0.48)^c^ 0.14 (0.06)^d^ 0.12 (0.20)^e^
Average months enrollment (SD)	33.3 (5.84)	32.9 (6.09)	33.4 (5.81)
Average number of notes (SD)^b^	132.00 (107.9)	235.50 (166.4)	117.88 (88.28)
Average number of characters (SD)^b^	165,766 (141,687)	290,913(215,529)	148,691 (118,534)

* Based on claims data from 2011 to 2013

^a^The Johns Hopkins ACG® System was used to generate the utilization rates using administrative claims data. ^b^Comparison of the non-frail versus frail population showed significant difference (*p* < .001) ^c^Unadjusted average and standard deviation. ^d^Adjusted for age and sex. ^e^Adjusted for age and sex, and comorbidity count

Abbreviations – ED: emergency department; F: female; IP: inpatient; M: male; N: count; SD: standard deviation

### Prevalence of frailty indicators

Clinicians referred to frailty status 7525 times in reference to 2202 patients. There was an average of 3 frailty mentions per patient, with a range of 1 to 59. The most common phrases identified were: “frail” with 6479 mentions, “frailty” with 671 mentions, “frail-appearing” with 171 mentions, “declining health” with 80 mentions, and “failing health” with 37 mentions. In an automated analysis of the text surrounding frailty descriptions, we found that at least 3077 mentions (40.8%) were in reference to the patient’s appearance or noted as part as the physical exam.

### Frailty status and geriatric syndromes

**Fig. 1:**
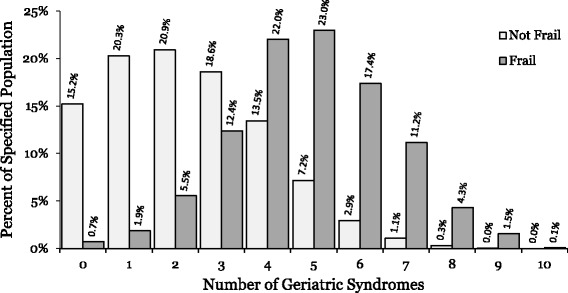
Number of Geriatric Syndromes among “Frail” and Non-frail Patients. Bar graph depicts the distributions of the “frail” (*n* = 2202) and non-frail (*n* = 16,139) populations based on the number of geriatric syndromes determined for each patient

We examined the relationship between frailty descriptions and the number of ten geriatric syndromes that were identified in each patient. We found that the “frail” patients had significantly more geriatric syndromes than non-frail patients: on average, “frail” patients had 4.85 ± 1.72 syndromes, while non-frail patients had only 2.35 ± 1.71 syndromes (*p* = .013) (Fig. 1). We also calculated the proportion of “frail” patients among all patients with a given number of syndromes: only 6.2% of all patients with less than 5 syndromes were “frail”, while 30.4% of all patients with exactly 5 syndromes were “frail”, and 52.0% of all patients with more than 5 syndromes were “frail”.

**Fig. 2:**
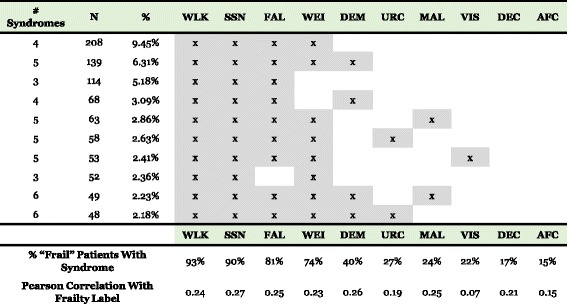
Ten Most Common Geriatric Syndrome Patterns and Correlations with Frailty Label. The top half of the figure gives the ten most common individual geriatric syndrome combinations observed in “frail” patients. N gives the number of “frail” patients with each exact syndrome combination, and the % column gives the percentage out of all 2202 “frail” patients. The bottom half of the figure gives the percentage of all “frail” patients with each individual syndrome, as well as the Pearson correlation of each individual syndrome with the frailty label. Abbreviations – AFC: absence of fecal control; DEC: decubitus ulcer; DEM: dementia; FAL: falls; MAL: malnutrition; N: count; SSN: lack of social support; URC: severe urinary control issues; VIS: visual impairment; WEI: weight loss; WLK: walking difficulty

We next examined geriatric syndrome patterns amongst “frail” patients. The most common individual syndrome pattern was a combination of walking difficulty, falls, weight loss and lack of social support, which was present in 208 (9.45%) patients (Fig. 2). Altogether, we found that 56.9% of the syndrome patterns observed in our “frail” population included this combination. Walking difficulty was the most commonly represented syndrome among patients described by their physician as “frail” (93%, Fig. 2). The least common syndrome among “frail” patients was absence of fecal control (15%, Fig. 2). A more detailed table describing the distribution of syndrome patterns is available in online Additional file 1.

We also assessed the Pearson correlation between a frailty label and each individual syndrome (Fig. 2). Visual impairment had the lowest correlation at 0.07, followed by absence of fecal control at 0.15 and severe urinary control issues at 0.19. Lack of social support and dementia had the highest correlations at 0.27 and 0.26, respectively. Correlations were higher between frailty labels and the combination of walking difficulty, falls, weight loss, and lack of social support (0.37; not shown in Fig. 2). Additionally, there was a high correlation between frailty labels and the number of geriatric syndromes in general (0.43; not shown in Fig. 2).

In an attempt to identify frailty using structured EHR fields, our expert panel identified three ICD9 codes that could represent frailty: senility without psychosis (797.x; includes frailty as an inclusion term), unspecified debility (799.3), and adult failure to thrive (783.7). Approximately 1100 patients had been assigned at least one of these ICD9 codes, with about half of these overlapping with patients already identified as frail in the free text. We ran additional analyses including patients with these ICD9 codes suggestive of frailty, and found similar results. These findings are described in online Additional file 1.

## Discussion

In this study, we developed and successfully implemented an NLP algorithm to identify geriatric syndromes and frail older adults in a large EHR-based dataset. Further, we examined associations and patterns of association between these geriatric syndromes and descriptions of frailty, in order to understand which clinical features drive a clinician to refer to a patient as “frail” in the EHR. We found that the most common constructs amongst “frail” patients were walking difficulty, lack of social support, falls, and weight loss; and, the least common were visual impairment, decubitus ulcers, and absence of fecal control (Fig. 2). Our analysis shows that almost half of frailty mentions in the EHR were made in reference to aspects of frailty that may be more likely to be observable on the patient’s appearance or the physical examination. Indeed, patients with weight loss or malnutrition will potentially appear thin and wasted, and those with walking difficulty and falls may have difficulty ambulating around the physician’s office or getting onto the examination table. These visual cues may suggest frailty to the observing provider.

It is unclear which features or geriatric syndromes inform a frailty assessment made by clinicians in day-to-day practice [20]. Previous work has found that bedside assessment of frailty by inpatient consultant cardiologists has poor agreement with the validated Reported Edmonton Frail Scale [21]. This assessment, however, was based solely on physical appearance and a brief discussion with the patient, in contrast to the clinicians in our study who engaged in a clinic visit with the patient and had access to their full medical record. While we were unable to compare with validated frailty measures in our study, it does seem that our providers were identifying a vulnerable population: patients described as “frail” had significantly more geriatric syndromes and higher rates of utilization than other patients (Fig. 1).

Notably, most of the higher correlated syndromes (e.g., walking difficulty, falls, malnutrition, and weight loss) align closely with Fried et al.’s criteria of syndromic frailty [6]. It is also of interest that the “frail” and “not frail” population curves depicted by the vertical bars in Fig. 1 cross around 3 syndromes, which parallels Fried et al.’s threshold of having at least 3 criteria to determine frailty.

Lack of social support and dementia were more highly correlated with frailty labels in our dataset than other syndromes. Although initial work focused on frailty as a physical syndrome related to muscle breakdown and weakness, more recently concepts like cognitive impairment and social factors have been incorporated into frailty definitions [22]. Our NLP algorithm was particularly valuable in this context as social factors are poorly coded in the structured fields [17, 23].

Visual impairment, absence of fecal control, and severe urinary control issues had overall lower correlations with frailty labels compared to other geriatric syndromes. Indeed, these syndromes are not included in many definitions of frailty [24]. For visual impairment, this could be because visual problems are commonly managed by ophthalmology as the primary provider, instead of primary care physicians or geriatricians who would be most concerned with frailty. The lower correlation for incontinence syndromes may be explained by under-recognition. The literature suggests that patients are less likely to report incontinence to their physician [25], general physicians are uncomfortable diagnosing incontinence [26], and incontinent patients are undertreated [27]. Incontinence may be multifactorial and influenced by another geriatric syndrome such as walking difficulty or dementia that impacts a patient’s ability to get to or properly use the toilet.

Although there were definite patterns in the correlations of individual syndromes and descriptions of frailty, the magnitude of all correlations was relatively low. There may be other clinical concepts that physicians value more in assessing frailty, which could be unearthed through machine learning and other NLP techniques [14, 28]. This reflects the multifactorial nature of geriatric syndromes like frailty, such that having any one geriatric syndrome does not push a patient above a provider’s frailty assessment threshold. This is supported by the fact that the correlation with number of syndromes and the correlation with a combination of syndromes were higher than the correlation of any individual syndrome.

Frailty has important implications in the healthcare delivery of older adults. Although EHRs using Read-CTV3 and SNOMED-CT coding terminologies have adopted specific coding for frailty [10], there is no standardized way to identify older adults at risk in EHRs using ICD coding for diagnostics, which are widely used in the U.S. The purpose of our study was not to propose a new model of frailty, but our results do show that older adults described by clinicians as frail were a vulnerable population with a higher geriatric syndrome burden and significantly higher healthcare utilization needs over our study period. Despite the lack of validated and standardized frailty indices built into ICD-based EHR systems, we demonstrate that at-risk patients can still be identified by using a pattern-based NLP algorithm on EHR system’s free text notes.

Our study has several limitations. First, our results are limited to Atrius Health’s population and providers and may not be generalizable to other older adults and providers in the **U.S.** Second, the NLP algorithm that we used to identify both patients with frailty mentions and the presence of the ten geriatric syndromes has nonzero false positive and false negative rates. Third, although geriatric syndromes have been linked to increased utilization in the older population, the consensus-derived subset and definitions of syndromes that we used have not specifically been validated or compared to existing frailty measures. Fourth, although we were given data about the type of each note and encounter, we do not know who specifically authored each note. Therefore, it is impossible to tell the qualifications or experience of the individuals who described a given patient as “frail”. Finally, we only had access to data stored in the EHR (structured and textual) prior to 2015, and neither had access to other components of the patient’s record including scanned forms, questionnaires or surveys, nor had ICD10 or other coding terminologies such as SNOMED to refine the selection process.

## Conclusion

Patients described as “frail” in the free text notes tended to have more geriatric syndromes and higher healthcare utilization than other patients, supporting that providers are identifying a vulnerable population and that they view frailty to be multifactorial. Geriatric syndromes such as walking difficulty, falls, weight loss and malnutrition were more highly correlated with clinician assessments of frailty than other geriatric syndromes. Future study is required to validate this clinical assessment of frailty against other frailty indices.

## Additional file

Additional file 1: Sample phrases, NLP methods, and additional results. (DOCX 36 kb)

## Abbreviations

ACO: Accountable care organization
AFC: Absence of fecal control
CTV3: Clinical terms version 3
DEC: Decubitus ulcer
DEM: Dementia
EHR: Electronic health record
F: Female
FAL: Falls
ICD: International classification of diagnosis
IP: Inpatient
M: Male
MAL: Malnutrition
N: Count
NLP: Natural language processing
OP: Outpatient
SD: Standard deviation
SNOMED: Systematized nomenclature of medicine – clinical terms
SSN: Lack of social support
URC: Severe urinary control issues
VIS: Visual impairment
WEI: Weight loss
WLK: Walking difficulty
