# Supplementary material for: Comparing clinician descriptions of frailty and geriatric syndromes using electronic health records: a retrospective cohort study
Source: BMC Geriatr. 2017 Oct 25;17:248. doi: 10.1186/s12877-017-0645-7 (PMC5657074; doi:10.1186/s12877-017-0645-7)
Supplement: Additional file 1: — Sample phrases, NLP methods, and additional results. (DOCX 36 kb) [file 12877_2017_645_MOESM1_ESM.docx]

# ONLINE SUPPLEMENT # S1

## Sample Phrases Identified by Manual Tagging

A panel of experts consisting of geriatricians and internists developed the initial list of phrases. The phrases were revised and amended after reviewing a select sample of patient records.

Table A1 – Sample Phrases Identified by Manual Tagging

| **Syndrome** | **Sample Phrases** | | | | |
| --- | --- | --- | --- | --- | --- |
| **AFC** | fecal incontinence | loses control of her bowels | bowel incontinence | fecal and urine leakage | incontinent of stool |
| **DEC** | sacral ulcer | stage 2-3 sacral decubitus | unstageable pressure wound | pressure ulcers | hip ulcer |
| **DEM** | patent has dementia | c/w dementia | decline of dementia | has been diagnosed with dementia | limited by dementia |
| **FAL** | has fallen | he fell on his buttocks | was dizzy and fell | with history of falling | admitted for fall |
| **MAL** | she is profoundly malnourished | protein-calorie malnutrition | feeding tube | nutritional supplements | Malnutrition |
| **SSN** | protective services | lives alone | social work intervention | referral for geriatric case manager | social isolation |
| **URC** | chronic urinary catheter | frequent changes of undergarments | double incontinence | atonic bladder | self- catheterization |
| **VIS** | functionally monocular | he is blind | legal blindness | Commission for the Blind | severe vision impairment |
| **WEI** | was down 10 pounds | weight loss significant | weight loss has become a problem | cachectic | progressive weight loss |
| **WLK** | rolling walker | gait abnormality | unsteadiness | difficulty with ambulation | cannot walk without help |
| **FR** | fragile, thin man | increasingly frail | appears to be extremely frail | Frail appearing | has been in failing health |

Abbreviations – AFC: absence of fecal control, DEC: decubitus ulcer, DEM: dementia, FAL: falls,
MAL: malnutrition, SSN: lack of social support, URC: severe urinary control issues,
VIS: visual impairment, WEI: weight loss, WLK: walking difficulty, FR: frailty;

Full list of phrases available upon request

# ONLINE SUPPLEMENT # S2

## Methodology and Evaluation of our Natural Language Processing Algorithm

An expert panel of geriatricians generated a list of seed phrases that would indicate the presence of each syndrome if one were found in the free text. A quasi-random selection of 185 patients was made from the whole population of 18,341 patients, using data from the claims and structured data to increase the chance there would be representative patients for each syndrome as well as patients without evidence of any syndrome. A team of three reviewers with medical background went through the 185 patient notes and tagged instances of each construct with a customized text editor, using the expert-generated seed phrases as a guide. This manually tagged corpus served as a gold standard against which we could compare our natural language processing (NLP) algorithm.

Using the phrases identified from manual tagging, a set of regular expressions was defined to automatically capture phrases related to each construct from the free text. Regular expressions use a special syntax to define flexible character-by-character patterns that can be used to query text. Another set of regular expressions was created to handle negation, diagnostic uncertainty, and to identify the subject of the syndrome. The regular expressions were run on the full population notes, and then were further refined through iterative manual review of randomly selected patients for each syndrome. We considered a patient to have a syndrome if there was at least one positive mention of that syndrome identified in the free text notes. Once finalized, we calculated the sensitivity and specificity of our algorithm against the 185 patient gold-standard and approximated false positive rates by manually reviewing approximately 100 random patient records for each syndrome from the full population (Table B1).

Note that advanced forms of NLP attempt to understand language through identifying grammar and parts of speech. Our approach uses character-based patterns that do not harness the full potential of true NLP algorithms.

Table B1 - Sensitivity, specificity and false positive rate of the NLP approach using
the 185 manually tagged patients (i.e., gold standard) and 100 random patients

|  | **185 Patients (Gold Standard)** | | **100 Random Patients** |
| --- | --- | --- | --- |
| *Syndrome* | *Sensitivity (%)* | *Specificity (%)* | *False Positive Rate (%)* |
| Absence of Fecal Control | 100 | 99.4 | 1.02 |
| Decubitus Ulcer | 100 | 100 | 4.04 |
| Dementia | 100 | 96.7 | 13.13 |
| Falls | 98.7 | 100 | 7.41 |
| Malnutrition | 87.5 | 100 | 6.12 |
| Lack of Social Support | 99 | 98.8 | 3.06 |
| Severe Urinary Control Issues | 100 | 99.4 | 8.08 |
| Visual Impairment | 94 | 100 | 5.05 |
| Weight Loss | 94.6 | 95.4 | 15.31 |
| Walking Difficulty | 96.5 | 98.6 | 2.04 |

# ONLINE SUPPLEMENT # S3

## Patterns and Prevalence of Individual Geriatric Syndromes in the “Frail” Population

Table C1 indicates the distribution of each geriatric syndrome within all observed geriatric syndrome combinations of the same number. The numbers in the cells represent the proportion of “frail” patients with a given number of syndromes who had each individual syndrome. Darker cells indicate more prevalent syndromes within each number-of-syndromes category.

For example, there were 273 “frail” patients who had exactly three geriatric syndromes. We found that 87% of these 273 patients had walking difficulty as one of their three geriatric syndromes, 82% had lack of social support, 63% had falls, 38% had weight loss, and so on.

Table C1 – Patterns and prevalence of Individual Geriatric Syndromes
in the “Frail” Population

|  | | **WLK** | **SSN** | **FAL** | **WEI** | **DEM** | **URC** | **MAL** | **VIS** | **DEC** | **AFC** | **N** |
| --- | --- | --- | --- | --- | --- | --- | --- | --- | --- | --- | --- | --- |
| **# Syndromes** | 0 | 0.00 | 0.00 | 0.00 | 0.00 | 0.00 | 0.00 | 0.00 | 0.00 | 0.00 | 0.00 | 16 |
|  | 1 | 0.29 | 0.27 | 0.20 | 0.17 | 0.00 | 0.02 | 0.00 | 0.02 | 0.00 | 0.02 | 41 |
|  | 2 | 0.70 | 0.39 | 0.36 | 0.39 | 0.07 | 0.03 | 0.02 | 0.04 | 0.00 | 0.01 | 122 |
|  | 3 | 0.87 | 0.82 | 0.63 | 0.38 | 0.09 | 0.03 | 0.04 | 0.11 | 0.02 | 0.01 | 273 |
|  | 4 | 0.95 | 0.94 | 0.79 | 0.68 | 0.24 | 0.09 | 0.09 | 0.13 | 0.03 | 0.05 | 485 |
|  | 5 | 0.99 | 0.99 | 0.89 | 0.84 | 0.45 | 0.23 | 0.20 | 0.20 | 0.12 | 0.10 | 506 |
|  | 6 | 0.99 | 1.00 | 0.95 | 0.92 | 0.58 | 0.42 | 0.39 | 0.31 | 0.25 | 0.20 | 383 |
|  | 7 | 1.00 | 0.99 | 0.96 | 0.96 | 0.75 | 0.64 | 0.54 | 0.37 | 0.47 | 0.32 | 246 |
|  | 8 | 1.00 | 0.99 | 0.98 | 1.00 | 0.83 | 0.84 | 0.62 | 0.56 | 0.55 | 0.63 | 94 |
|  | 9 | 1.00 | 1.00 | 1.00 | 1.00 | 0.88 | 0.91 | 0.82 | 0.59 | 0.94 | 0.85 | 34 |
|  | 10 | 1.00 | 1.00 | 1.00 | 1.00 | 1.00 | 1.00 | 1.00 | 1.00 | 1.00 | 1.00 | 2 |

Abbreviations – N: number of patients with a given number of syndromes, AFC: absence of fecal control, DEC: decubitus ulcer, DEM: dementia, FAL: falls, MAL: malnutrition, SSN: lack of social support, URC: severe urinary control issues, VIS: visual impairment, WEI: weight loss, WLK: walking difficulty, FR: frailty

# ONLINE SUPPLEMENT # S4

## Results Including Patients with Structured Frailty Codes

In an attempt to analyze frailty in the structured fields, our expert panel identified three ICD9 codes that could represent frailty: senility without psychosis (797.x), unspecified debility (799.3), and adult failure to thrive (783.7). Approximately 1100 patients had been assigned at least one of these ICD9 codes, and we ran additional analyses including these patients. In total, 2784 patients had either a mention of frailty in EHR’s free text or an encoded frailty ICD9 code (i.e., any-frail population), from whom 582 patients did not have any mention of frailty in free text, 1679 only had it in free text, and the rest (n = 523) had it in both free text and encoded fields. Following tables and figures are the results of the same analysis of the manuscript but for this expanded population denominator of frail older adults (n = 2784).

Table D1 – Population Demographics and Healthcare Utilization

| **Demographics/Utilization**** | **Full Population** | **“Frail” Population** |
| --- | --- | --- |
| Population (N) | 18,341 | 2,784 |
| Age in years (SD)* | 75.9 (7.5) | 81.8 (7.1) |
| Sex (%)* | F 10,806 (58.9) M 7,535 (41.1) | F 1,832 (65.8)  M 952 (34.1) |
| Average comorbidity count (SD)* | 9.16 (4.72) | 13.13 (5.17) |
| Average ingredients (SD)* | 12.57 (7.73) | 16.63 (8.95) |
| Average IP events (SD)* | 0.86 (1.59) | 2.30 (2.60) |
| Average ED events (SD)* | 0.62 (1.26) | 1.27 (2.07) |
| Average readmission events (SD)* | 0.15 (0.65) | 0.52 (1.29) |
| Average months enrollment (SD)* | 33.3 (5.84) | 32.6 (6.37) |
| Average number of notes (SD)* | 132.00 (107.9) | 222.6 (160.8) |
| Average number of characters (SD)* | 165,766 (141,687) | 273,041 (210,502) |

Demographic and healthcare utilization for the full population of 18,341 patients and the 2,784 any-frail patients. This includes all available claims data for a patient during our study period from 2011 to 2013.

* Comparison of the full versus any-frail population showed significant difference (p<.001);
**The Johns Hopkins ACG System was used to generate the utilization rates using administrative claims data.

Abbreviations – N: count, F: female, M: male, SD: standard deviation,
IP: inpatient, ED: emergency department

Figure D1 – Number of Geriatric Syndromes among Any-Frail and Non-frail Patients

*Bar graph depicts the distributions of the any-frail (n=2,784) and non-frail (n=15,557) populations
based on the number of geriatric syndromes determined for each patient.*

Figure D2 – Common Geriatric Syndrome Combinations and Correlations with Frailty Label

| **# Syndromes** | **N** | **%** | **WLK** | **SSN** | **FAL** | **WEI** | **DEM** | **URC** | **MAL** | **VIS** | **DEC** | **AFC** |
| --- | --- | --- | --- | --- | --- | --- | --- | --- | --- | --- | --- | --- |
| 4 | 267 | 9.59% | x | x | x | x |  |  |  |  |  |  |
| 5 | 183 | 6.57% | x | x | x | x | x |  |  |  |  |  |
| 3 | 134 | 4.81% | x | x | x |  |  |  |  |  |  |  |
| 4 | 82 | 2.95% | x | x | x |  | x |  |  |  |  |  |
| 5 | 82 | 2.95% | x | x | x | x |  |  | x |  |  |  |
| 3 | 77 | 2.77% | x | x |  | x |  |  |  |  |  |  |
| 6 | 70 | 2.51% | x | x | x | x | x | x |  |  |  |  |
| 5 | 69 | 2.48% | x | x | x | x |  | x |  |  |  |  |
| 5 | 66 | 2.37% | x | x | x | x |  |  |  | x |  |  |
| 6 | 60 | 2.16% | x | x | x | x | x |  | x |  |  |  |
|  |  |  | **WLK** | **SSN** | **FAL** | **WEI** | **DEM** | **URC** | **MAL** | **VIS** | **DEC** | **AFC** |
| **% Frail Patients With Syndrome** | | | 92% | 90% | 79% | 74% | 40% | 27% | 21% | 23% | 17% | 14% |
| **Pearson Correlation With Frailty Label** | | | 0.26 | 0.31 | 0.26 | 0.26 | 0.3 | 0.21 | 0.27 | 0.07 | 0.24 | 0.16 |

*The top half of the figure gives the ten most common individual geriatric syndrome combinations observed in our population of any-frail patients. N gives the number of any-frail patients with each exact syndrome combination, and the % column gives the percentage out of all 2,784 any-frail patients.*

The bottom half of the figure gives the percentage of all any-frail patients with each individual syndrome, as well as the Pearson correlation of each individual syndrome with the frailty indicator.

Abbreviations – WLK: walking difficulty, SSN: lack of social support, FAL: falls,
WEI: weight loss, DEM: dementia, MAL: malnutrition, URC: severe urinary control issues
DEC: decubitus ulcer, AFC: absence of fecal control, VIS: visual impairment, N: count
